# Optimizing QoS and security in agriculture IoT deployments: A bioinspired Q-learning model with customized shards

**DOI:** 10.1016/j.heliyon.2024.e24224

**Published:** 2024-01-09

**Authors:** Sonali Mahendra Sonavane, G.R. Prashantha, Pranjali Deepak Nikam, Mayuri A V R, Jyoti Chauhan, Sountharrajan S, Durga Prasad Bavirisetti

**Affiliations:** a G H Raisoni College of Engineering and Management, Pune, Maharashtra, India; b Jain Institute of Technology, Davangere, Karnataka, India; c Anantrao Pawar College of Engineering and Research, Pune, Maharashtra, India; d School of Computing Science and Engineering, VIT Bhopal University, Sehore, Madhya Pradesh, India; e Department of Computer Science and Engineering, Amrita School of Computing, Amrita Vishwa Vidyapeetham, Chennai, India; f Department of Computer Science, Norwegian University of Science and Technology (NTNU), Trondheim, Norway

**Keywords:** Blockchain, AIoT, QoS, Security, Sharding, Custom, PoP, Mayfly, Bacterial, Foraging, Delay, Throughput, Attacks

## Abstract

Agriculture Internet of Things (AIoTs) deployments require design of high-efficiency Quality of Service (QoS) & security models that can provide stable network performance even under large-scale communication requests. Existing security models that use blockchains are either highly complex or require large delays & have higher energy consumption for larger networks. Moreover, the efficiency of these models depends directly on consensus-efficiency & miner-efficiency, which restricts their scalability under real-time scenarios. To overcome these limitations, this study proposes the design of an efficient Q-Learning bioinspired model for enhancing QoS of AIoT deployments via customized shards. The model initially collects temporal information about the deployed AIoT Nodes, and continuously updates individual recurring trust metrics. These trust metrics are used by a Q-Learning process for identification of miners that can participate in the block-addition process. The blocks are added via a novel Proof-of-Performance (PoP) based consensus model, which uses a dynamic consensus function that is based on temporal performance of miner nodes. The PoP consensus is facilitated via customized shards, wherein each shard is deployed based on its context of deployment, that decides the shard-length, hashing model used for the shard, and encryption technique used by these shards. This is facilitated by a Mayfly Optimization (MO) Model that uses PoP scores for selecting shard configurations. These shards are further segregated into smaller shards via a Bacterial Foraging Optimization (BFO) Model, which assists in identification of optimal shard length for underlying deployment contexts. Due to these optimizations, the model is able to improve the speed of mining by 4.5%, while reducing energy needed for mining by 10.4%, improving the throughput during AIoT communications by 8.3%, and improving the packet delivery consistency by 2.5% when compared with existing blockchain-based AIoT deployment models under similar scenarios. This performance was observed to be consistent even under large-scale attacks.

## 1 Introduction

The “Internet of Things” (IoT) is a paradigm in which a large number of tangible objects are networked using wired or wireless technologies and then linked to the Internet so that they can be accessed from anywhere [[1], [2], [3]]. Use of Trust Aggregation Certificate-Based Authentication Scheme (TACAS) verifies these facts. This linking of the objects to the internet makes it possible for users to access the objects from any location. IoT applications have become increasingly widespread across a variety of sectors over the course of the last several years, including the manufacturing industry, the home automation industry, the transportation industry, and the healthcare industry [[4], [5], [6]]. Large-scale Agriculture Internet of Things (AIoT) infrastructures are often built, deployed, and managed independently by a number of different companies in the modern day. The vast majority of them are hosted in the cloud and rely on centralized communication protocols [[7], [8], [9]]. This implies that all devices are identified, connected, and approved by means of an architecture that is powered by a massively powerful cloud server. Alternately, the growing magnitude, complexity of network and cloud infrastructures is contributing to an increase in the cost of centralized AIoT systems [[10], [11], [12]]. An additional layer of complexity is introduced whenever there is a growing need for data to be interoperable, immutable, and verifiable when it is being traded across AIoT networks that include several parties. Because of the possibility that data supplied by specific Agriculture partners may have been tampered with or altered by attackers or the owner of the data in conventional AIoT networks, such data may not be trustworthy. In the context of AIoT networks, there is widespread consensus that data verification methods should be implemented in different scenarios [[13], [14], [15]].

The use of blockchain technology is a significant competitor in the race to fulfil the need for services that are both unchangeable and verifiable [[16], [17], [18]]. A distributed, block-based ledger that operates on a decentralized peer-to-peer network is referred to as blockchain. It eliminates the need for a reliable third party to mediate transactions and makes it possible for parties to do business without having to rely on the confidence in one another [19,20]. The management of unidentified devices and processes, as well as device-to-device transactions and communications, would be considerably simplified by the integration of blockchain technology into AIoT systems. Crypto-contracts between AIoT devices (such as self-executing and self-enforcing protocols) may be posted on blockchain as smart contracts [[21], [22], [23], [24]] and implemented automatically, which will significantly increase the efficacy of transactional processes. Because of the extremely limited resources available in AIoT networks and the requirement of the blockchain technology that each participant keep an identical copy of the blockchain to ensure consistency, the blockchain technology cannot be easily integrated into the AIoT systems that are currently in use. One of the most well-known applications that make use of blockchain technology is bitcoin. Even though it is seldom updated, the Bitcoin blockchain already holds more than 190 GB of datasets & samples [25,26]. However, only a very small amount of this information is required for the functioning of the financial system. The great majority of large-scale Internet of Things (IoT) devices produce data that is just too massive to be stored locally. This is in contrast to the cryptocurrency Bitcoin, whose blocks can be retained locally. As a result, it is of the utmost need to develop a new method that is both scalable and capable of storing blockchain datasets & samples [[27], [28], [29]]. This may be accomplished by removing redundant layers from the current system. This section explains how a bio-inspired Q-Learning model was developed in order to enhance the quality of service (QoS) of AIoT installations by making use of specialized shards. In Section 4, we analyse the efficacy of the model in a variety of attack situations by comparing its results to those of existing methodologies. This allows us to determine how well the model performs. This article comes to a close with some concluding thoughts on the model that was proposed as well as some ideas for enhancing the model's use in real world scenarios.

Many existing security models for AIoT deployments rely on blockchain technology, which can introduce significant complexity. This complexity can hinder the practical implementation of such models, making them less suitable for large-scale agriculture IoT networks. To identified challenges with AIoT deployments are Complexity of Existing Blockchain-Based Models, the Delay and energy consumption, Scalability Limitations, Uniform Shard Configuration, Lack of Optimization Models.

The main objective of the work is enhancing the Quality of Service and security in Agriculture Internet of Things (AIoT) deployments. We also aim to minimize delay and energy consumption associated with security and consensus mechanisms in AIoT deployments. The paper aims to improve the scalability of the proposed model under real-time scenarios, ensuring that it can accommodate the increasing demands of AIoT deployments without compromising performance. It also focuses on optimizing various aspects of the proposed bioinspired Q-Learning model with customized shards. In response to these challenges and deficiencies, the paper proposes a novel bioinspired Q-Learning model with customized shards that aims to simplify AIoT security, reduce delays and energy consumption, enhance scalability, improve miner selection based on trust metrics, optimize block addition through PoP-based consensus, and customize shard configurations using optimization models like MO and BFO. These improvements are designed to address the limitations of existing solutions and enhance the performance and security of AIoT deployments in agriculture.

## 2 Issues with cloud based AIoTs

**Fig. 1:**
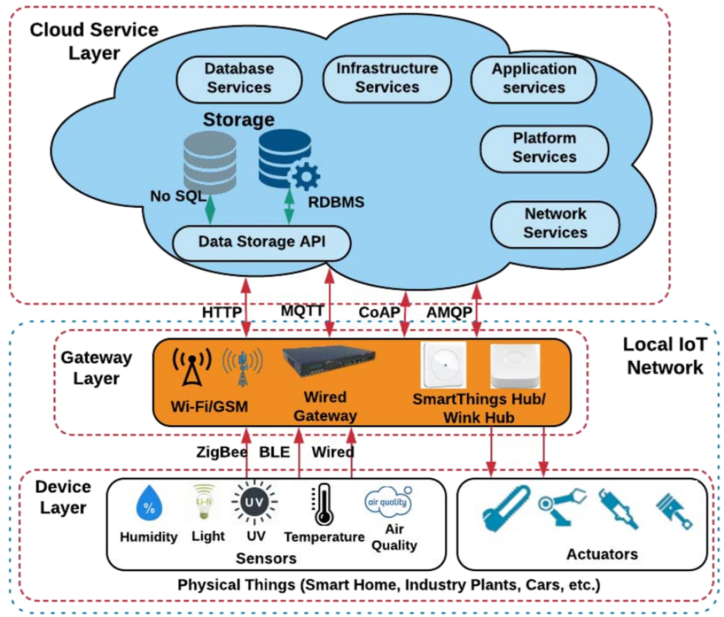
Cloud AIoT infrastructure deployments.

The fundamental components of a cloud-based AIoT architecture are depicted in Fig. 1, wherein the device layer, gateway layer, and cloud service layer include these major components. The device layer of the AIoT may include anything from extremely effective microcontrollers to sophisticated central processing units. These devices may connect to the gateway layer via a number of wired and wireless networking protocols, including ZigBee, BLE, Ethernet, and others.

The majority of enterprises and organizations construct their own unique hub-layer ports to connect to the cloud, collect data, and manage local Internet of Things (AIoT) networks [7,8]. Since these specialized gateways are often used as part of the AIoT infrastructure, there has been a boom in the number of “stovepipe” solutions [9]. As a consequence of this change, interoperability concerns are heightened even more. Because of variations in networking protocols, data formats, and other considerations, interoperability concerns impede the exchange of data and services supplied by one firm with devices created by another. In addition, security measures are often kept confidential or are not made available to the general public. To aid clarity and conciseness, the word “IoT” will serve as a substitute for “AIoT” for the whole of this discussion. The two components of locally-based Internet of Things networks are the device layer and the gateway layer. The vast majority of Internet of Things devices are deployed in uncontrolled settings in order to collect and monitor data about connected physical or digital objects. The amount of storage capacity they have, the processing power they have, and the rates at which they can transfer data are all constrained [10]. In addition, the devices and networking protocols used by these systems are quite diverse. The vast array of devices that comprise the AIoT makes system integration more complicated. IoT devices must be able to interact with one another so that customers can comprehend the syntax and semantics of a range of devices [11]. In local Internet of Things networks, centralized storage solutions are often utilized to manage IoT data, as opposed to distributed or local management options. When using a centralized storage system, it is the responsibility of the local gateway to capture data and then transfer it to a centralized local storage facility so that the data may be preserved safely. The proposed approach to IoT architecture involves storing data in a single, easily accessible location, such as a data center or archivist's office. This centralized storage allows faster access to the most recent data without requiring cloud storage. The data engine transforms unstructured data into representations that can be used, and in the blockchain-based architecture, raw data is transformed into transactions, which are encrypted before being transferred to the cloud when necessary. Key management and security procedures are provided by the data engine to ensure data transmission safety. The blockchain-based architecture serves as a peer-to-peer node within the blockchain overlay network, providing proxy services, routing information, authentication, and multicast group management [12]. It also functions as a sink for the local IoT network. The cloud service layer provides cloud-based services to manage data from local IoT networks, including database and application services.

Blockchain technology has the potential to significantly enhance the value of existing IoT capabilities by incorporating cryptographic techniques and addressing security and trustworthiness issues in large-scale IoT systems [13]. It is likely that blockchain technology will be utilized to record sensor readings from the internet of things in order to prevent data manipulation and fabrication. Using a blockchain, IoT devices may be able to create mutual trust without the need for a third party. Blockchain's distributed ledger design eliminates a weak link in the Internet of Things (IoT) ecosystem and safeguards the data produced and stored by IoT devices. This is achieved by eliminating the system's single point of failure. Blockchain technology facilitates peer-to-peer (P2P) communication by removing several technical barriers and promoting device autonomy via the use of smart contracts, individual identities, and data integrity. IoT devices have the ability to generate a large amount of data in a short amount of time, necessitating the storage of both the data hash and the actual data. Costs associated with maintaining a blockchain have the potential to skyrocket over time owing to the increasing storage and bandwidth requirements of all nodes as the network grows. In this article, we examine the topic of implementing blockchain technology into the cloud-based architecture of the internet of things in an effort to solve the issue of limited storage space. Bitcoin is one of the most successful applications of the blockchain, which is one of the various emerging research concepts [14,15]. In addition to the block's content, the block contains the block's header. Even though the block header is a fairly little file, the auditors are needed to download the whole Bitcoin blockchain. This is a big issue with the witnessing approach utilized for Bitcoin transactions at now. As of November 2022, the Bitcoin blockchain included around 2.5 Petabytes of data, and it continues to grow at a pace of 85 gigabytes per year [14,16,17]. It is difficult, to download and save the whole blockchain on IoT gateways that have limited storage capacity and bandwidth.

## 3 Literature review

The Internet of Things (IoT) has revolutionized the way devices communicate with each other, enabling new applications and services. In the context of Agriculture IoT (AIoT), the deployment of IoT devices has been instrumental in enhancing operational efficiency, reducing downtime, and improving overall productivity. However, ensuring reliable and high-quality service delivery remains a challenge in AIoT deployments, as they typically operate in harsh and dynamic environments. To address the challenges of QoS in AIoT deployments, several methods have been proposed in the literature. In this literature review, we discuss some of the most commonly used methods for enhancing the QoS of AIoT deployments.

• Edge computing and Reinforcement Learning (RL) [[30], [31], [32]]: Edge computing has emerged as a promising approach for improving QoS in AIoT deployments. By bringing computing resources closer to the edge of the network, edge computing reduces latency and improves the reliability of data transmissions [[33], [34], [35]]. Furthermore, edge computing allows for real-time processing of data enabling quick decision-making and reducing the risk of downtime levels.
• Quality of Service (QoS) mechanisms [[36], [37], [38], [39]]: QoS mechanisms, such as traffic shaping and prioritization, can be used to allocate network resources via Secure Many-to-Many Authentication and Key Agreement (SMAA) in a way that ensures high-quality service delivery levels [[40], [41], [42]]. By prioritizing critical data traffic over non-critical traffic, QoS mechanisms can reduce network congestion and ensure reliable delivery of critical datasets & samples [43,44].
• Redundancy and fault-tolerance [45,46]: In AIoT deployments, it is essential to have redundant systems and fault-tolerant architectures in place to ensure that critical systems continue to operate in the event of a failure. Redundancy can be achieved through backup systems, while fault-tolerance can be achieved through techniques such as replication and load balancing.
• Protocol selection [[47], [48], [49]]: The choice of communication protocol can have a significant impact on QoS in AIoT deployments. For example, protocols such as MQTT and CoAP are designed for low-power and low-bandwidth devices, while protocols such as TCP and HTTP are better suited for high-bandwidth devices.
• Predictive maintenance [50]: Predictive maintenance is a method of using data analytics and machine learning to predict when equipment will fail for different scenarios. By identifying potential failures before they occur, predictive maintenance can reduce downtime and improve the overall reliability of AIoT deployments [51,52].
• Cryptographic attacks: Various attacks like Masquerading & authentication attack, dictionary attack, Brute Force attack, fault attack, Side chain attacks are studied for checking the network performance of model [[53], [54], [55]].

## 4 Proposed design of an efficient Q-learning bioinspired model for enhancing QoS of agriculture IoT deployments via customized shards

**Fig. 2:**
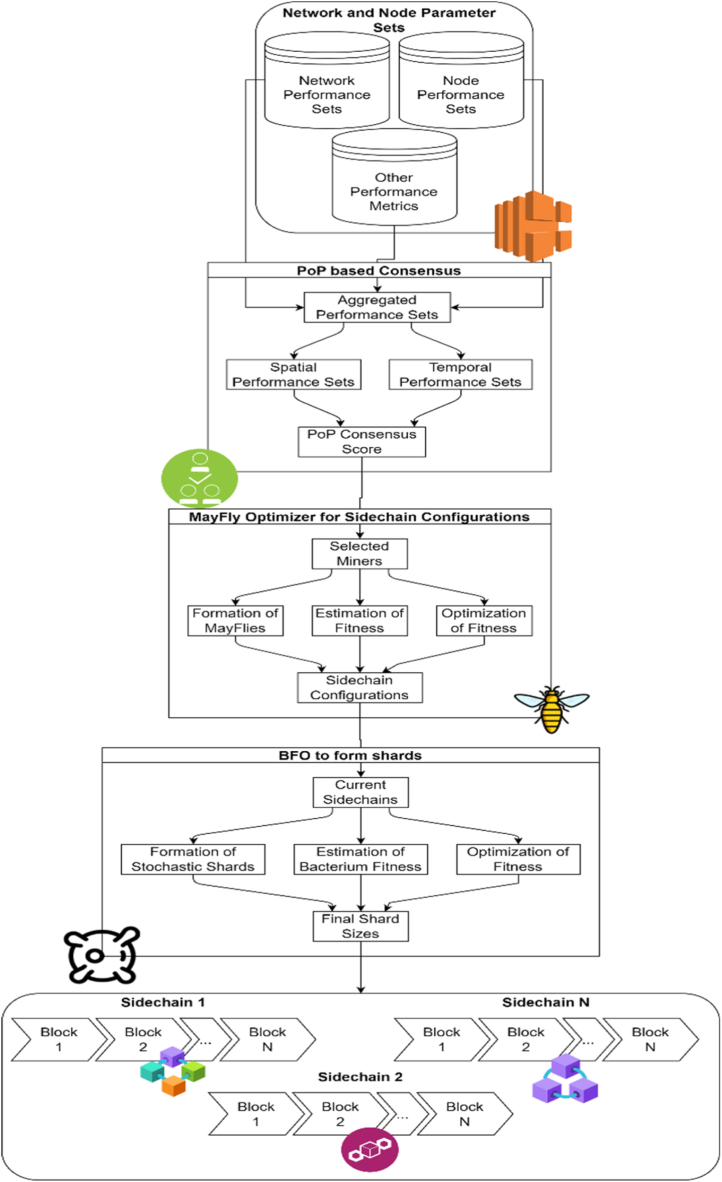
Design of the proposed model for optimization of blockchain deployments.

As per the review of existing models used to improve QoS of IoT deployments, it can be observed that existing security models that employ blockchains are either incredibly complex, or they necessitate substantial delays and have a greater energy footprint for larger networks. In addition, the efficacy of these models is directly dependent on consensus-efficiency and miner-efficiency, limiting their scalability in real-time scenarios. This section discusses the design of an efficient Q-Learning bioinspired model for enhancing the QoS of AIoT deployments via customized shards in order to overcome these limitations. As per flow of the model in Fig. 2, it can be observed that the proposed model collects initial temporal data about the deployed AIoT Nodes and continuously updates individual recurring trust metrics. A Q-Learning process uses these trust metrics to identify miners who can participate in the block-addition procedure. The blocks are added using a Proof-of-Performance (PoP)-based consensus model that employs a dynamic consensus function based on the temporal performance of miner nodes. Each shard is deployed based on its deployment context, which determines the shard-length, hashing model used for the shard, and encryption technique used for these shards. This is made possible by a Mayfly Optimization (MO) Model that selects shard configurations based on PoP scores. Using a Bacterial Foraging Optimization (BFO) Model, these shards are further subdivided into smaller shards, which facilitate the determination of optimal shard length for underlying deployment contexts.

As per the flow of this model, it can be observed that the model collects a wide variety of node-level performance parameters, network-level performance parameters, and temporal performance parameters in order to deploy PoP based consensus. This consensus model initially estimates a PoP Q-Learning Score (PQS) via Equation (1), which is a fusion of temporal and spatial performance metrics. This score combines temporal Packet Delivery Ratio (PDR), temporal Throughput (THR), temporal Delay (D) during communication, and temporal Energy (E) consumed during communication with instantaneous distance (d), and residual energy metrics (e), which assist in comprehensive evaluation of miner nodes under real-time scenarios.

(1) $$PQS\left(i,j\right)=\frac{e\left(i\right)}{NC\left(i,j\right)*d\left(i,j\right)}\sum_{i=1}^{NC\left(i,j\right)}\frac{\mathrm{Max}\left(D\right)}{D\left(i\right)}+\frac{\mathrm{Max}\left(E\right)}{E\left(i\right)}+\frac{PDR\left(i\right)}{100}+\frac{THR\left(i\right)}{\mathrm{Max}\left(THR\right)}$$

where, NC(i,j) represents number of temporal communications between nodes i&j which were performed during previous mining operations. The temporal delay is estimated via Equation (2),

(2) D=t(complete)−t(start)

where, t(complete),t(start) are the timestamps of completion and starting for these communications. Similarly, the energy consumes is estimated via Equation (3),

(3) E=E(start)−E(complete)

where, E(start)&E(complete) are the initial & completion residual energy levels of miner nodes. The throughput is estimated via Equation (4),

(4) $$THR=\frac{Rx\left(P\right)}{D}$$

where, Rx(P) are total number of successfully mined packet requests, which are also used during estimation of PDR levels via Equation (5),

(5) $$PDR=\frac{Rx\left(P\right)}{Tx\left(P\right)}$$

where, Tx(P) are total number of transmitted mining requests during the temporal communication requests. The distance between miners is estimated via Equation (6),

(6) $$d\left(i,j\right)=\sqrt{{\left(x\left(i\right)-x\left(j\right)\right)}^{2}+{\left(y\left(i\right)-y\left(j\right)\right)}^{2}.}$$

After estimating this score for all miner nodes, a PoP threshold is estimated via Equation (7),

(7) $$PoP\left(th\right)=\frac{1}{N{\left(M\right)}^{2}}\sum_{i=1}^{N\left(M\right)}\sum_{j=1}^{N\left(M\right)}PQS\left(i,j\right)$$

where, N(M) are Number of Miners that are contesting in the mining consensus process. Node pairs that satisfy Equation (8) as selected for the mining consensus process,

(8) PQS(i,j)>PoP(th)*F(MO)

where, F(MO) is a tuning factor, and is estimated via Mayfly Optimization process. Based on these operations, a set of miner nodes are selected that can take part in consensus. Block addition decisions taken by these nodes assist the network to optimize QoS, while maintaining higher security levels. Performance of this model is further tuned by Mayfly optimizer, which assists in selection of context-aware blockchain entities. These include,

● Hashing Algorithm used during linking the blocks
● Encryption Algorithm used for securing the blocks
● PoP Consensus tuning factor, which is used to optimize selection of miners

The Mayfly optimizer works as per the following operations,

● A set of NM Mayflies are initially generated, where each Mayfly consists of a combination of Hashing Technique, Encryption Technique and PoP tuning factor, which are estimated via Equations (9), (10), (11) as follows,

(9) H=STOCH(1,NH)

(10) E=STOCH(1,NE)

(11) F(MO)=STOCH(LM,1)

where, STOCH is a stochastic operator, NH&NE are the number of hashing & encryption models supported by underlying blockchains, while LM represents the learning metric for Mayfly optimization process.

● In context of this text, hashing models supported are MD5,SHA1,SHA256,SHA512, while encryption models supported are variants of different ECC curves.
● Using these parameters, a set of N dummy blocks are added to the blockchain, and Mayfly fitness is estimated via Equation (12),

(12) $$fm=\frac{1}{N}\sum_{i=1}^{N}d\left(hashing,i\right)+d\left(encryption,i\right)+d\left(mining,i\right)$$

where, d represents delay levels for different hashing, encryption & mining operations.

● This process is repeated for NM Mayflies, and a fitness threshold is estimated via Equation (13),

(13) $$fth=\frac{1}{NM}\sum_{i=1}^{NM}fm\left(i\right)*LM$$

● Mayflies with fm>fth are regenerated, while others are passed directly to the next set of iterations.
● After repeating this process for NI Iterations, Mayfly with lowest fitness is selected for context-based blockchain modelling process.

Values of H,E&F(MO) are used to add new blocks to the blockchain, which assists in reducing mining delay, while maintaining higher security levels. After addition of each block, a sidechain decision threshold is estimated via Equation (14), which assists the model to activate sidechain decision making process.

(14) $$SC\left(th\right)=\frac{d\left(Mine,New\right)*e\left(Mine,New\right)}{d\left(Mine,Prev\right)*e\left(Mine,Prev\right)}$$

where, d(Mine)&e(Mine) represents the delay & energy needed during mining operations. An iterative value of SC(th)>4, indicates that the current sidechain configuration might be taking exponentially large delay & consuming larger number of energy units to add new blocks. In such a case, the proposed model uses a Bacterial Foraging Optimizer (BFO) to form new sidechain configurations. The BFO selections optimal sidechain lengths, which assist in improving network QoS even under higher traffic scenarios. The BFO Model works as per the following operations,

● A set of NB Bacterium solutions are generated, where each solution selects a sidechain length N(SC) via Equation (15),

(15) N(SC)=STOCH(LB*N(B),N(B))

where, LB is the bacterium learning rate, and N(B) represents total number of blocks in the current configuration of sidechains.

● Using this length of sidechain, current blockchain is segregated into two parts, and the part with lower number of blocks is used for the evaluation process.
● In this process, a set of N dummy blocks are added to the smaller chain, and Bacterium fitness is estimated via Equation (16),

(16) $$fb=\frac{1}{N}\sum_{i=1}^{N}\left(\frac{d\left(mining,i\right)}{\mathrm{Max}\left(d\right)}+\frac{e\left(mining,i\right)}{\mathrm{Max}\left(e\right)}\right)*\frac{N\left(M,i\right)}{\mathrm{Max}\left(N\left(M\right)\right)}$$

where, d&e represents the delay & energy needed during mining, while N(M) represents number of miners that took part in the consensus process.

● This process is repeated for NB Bacterium, and a set of upper and lower thresholds are estimated via Equations (17), (18) as follows,

(17) $$f\left(Lower\right)=\sum_{i=1}^{NB}\begin{array}{c} fb\left(i\right)*\frac{LB}{2*NB} \end{array}$$

(18) $$f\left(Upper\right)=\sum_{i=1}^{NB}fb\left(i\right)*\frac{LB*2}{NB}$$

● Bacterium that satisfy Equation (19) as passed directly to the next iteration, while others are modified via Equations (15), (16)) in the current set of iterations.

(19) f(b)>f(Lower)ANDf(b)<f(Upper)

● This process is repeated for a set of NI Iterations, and new sidechain configurations are generated via these operations.

At the end of NI Iterations, Bacteria particle that satisfies Equation (20) is selected to form new sidechains.

(20) $$f\left(b\right)≅\frac{f\left(Upper\right)+f\left(Lower\right)}{2}$$

Based on the sidechain length identified by this Bacteria, current blockchain is segregated into 2 sidechains. The sidechain with lower length is used to add new blocks, while sidechain with higher length is merged with existing blockchain, and is used for archival purposes. Due to this, the model is able to reduce delay & energy needed for mining, while maintaining higher throughput and PDR levels. Performance of this model is evaluated under different conditions and discussed in the next section of this text.

### 4.1 Application and functioning of the Q-learning bioinspired model with customized shards

#### 4.1.1 Data collection and trust metrics

The Q-learning bioinspired model begins by collecting temporal information about the AIoT nodes deployed in the agricultural network. This information can include node performance data, historical behavior, and communication patterns. Trust metrics are continuously updated for each node based on its behavior over time.

#### 4.1.2 Miner selection process

The trust metrics play a pivotal role in the miner selection process. Nodes with higher trust scores are considered more reliable and are more likely to be selected as miners. Q-learning algorithms are employed to assess the trust metrics of nodes and determine which nodes are best suited to participate in the block-addition process.

#### 4.1.3 Proof-of-performance (PoP) consensus model

In PoP, miners must demonstrate consistent and high-quality performance over time to be eligible to add blocks to the blockchain. The performance criteria can include low latency, high throughput, and efficient resource utilization. The dynamic consensus function adapts to changing network conditions and miner performance, ensuring that only nodes meeting the PoP criteria can contribute to the blockchain.

#### 4.1.4 Customized shards

Customized shards are a key feature of this model. Each shard is tailored to its specific context of deployment, considering factors such as the nature of IoT devices, communication patterns, and geographic location. The customization process determines the shard's length, hashing model used, and encryption technique applied. This ensures that resources are allocated optimally for each shard's unique requirements.

#### 4.1.5 Mayfly optimization (MO) model

The MO model leverages PoP scores to select shard configurations. It uses these scores to determine which shard parameters will yield the best performance results for each specific shard. By utilizing PoP, scores in this manner, the MO model optimizes the efficiency of shard configurations, improving the overall performance of the network.

#### 4.1.6 Bacterial foraging optimization (BFO) model

The BFO model further enhances shard optimization by segregating shards into smaller, more finely-tuned sub-shards. This process helps identify the optimal shard length for each deployment context. By adapting shard lengths to the unique requirements of each context, the BFO model contributes to improved resource allocation and network performance.

### 4.2 Contrasting proof-of-performance (PoP) consensus models

The Proof-of-Performance (PoP)-based consensus model is a novel approach to achieving consensus in blockchain networks. To understand why PoP is considered superior, let's contrast it with Proof-of-Work (PoW) and Proof-of-Stake (PoS). **Energy Efficiency**: PoP significantly reduces energy consumption as it focuses on selecting miners based on their consistent performance rather than computational work. **Low Latency**: PoP dynamically adjusts to changing network conditions, reducing latency by allowing only high-performing miners to participate. **Security**: PoW is secure against attacks, but it requires a substantial amount of computational resources to maintain security. Whereas PoP enhances security by ensuring that only trustworthy and high-performing nodes can contribute to the blockchain. **Scalability**: PoP is more scalable than PoW because it does not rely on resource-intensive computations, making it suitable for large-scale networks. PoS has faced criticisms regarding centralization.

### 4.3 Sharding analysis

Sharding is a technique used in blockchain and distributed ledger technologies to improve scalability and overall network performance. The rationale behind creating shards is to address the limitations of monolithic blockchains, enhance scalability, reduce latency, optimize resource utilization, improve security, and customize the blockchain network for specific use cases. The performance impact of sharding is significant, leading to increased transaction throughput, reduced latency, efficient resource usage, enhanced security, and adaptability to a wide range of applications. Sharding is a crucial technique for the evolution of blockchain technology, allowing it to meet the demands of diverse and complex use cases.

## 5 Result evaluation & comparison

Initially, the proposed model gathers historical data on the AIoT Nodes in operation, and it then continuously updates various repeating trust metrics. In order to determine which miners are qualified to add blocks, a Q-Learning process uses these trust metrics. A new Proof-of-Performance (PoP) based consensus model employs a consensus function that changes based on the current and past performance of miner nodes to add new blocks to the blockchain. Customized shards facilitate the PoP consensus by varying the shard-length, hashing model used for the shard, and encryption technique used by each shard based on its deployment context. A Mayfly Optimization (MO) Model helps with this process by utilizing PoP scores to decide on shard configurations. The optimal shard length for a given deployment scenario can be determined with the help of a Bacterial Foraging Optimization (BFO) Model, which further divides these shards into even smaller shards. To evaluate QoS performance of the model, a set of different metrics including energy consumed during mining (E), delay needed during mining (D), throughput (T), PDR, and Jitter (J) during mining were evaluated via Equations (21), (22), (23), (24), (25) as follows,

(21) $$E=\frac{1}{N\left(M\right)}\sum_{i=1}^{N\left(M\right)}E\left(start,i\right)-E\left(complete,i\right)$$

(22) $$D=\frac{1}{N\left(M\right)}\sum_{i=1}^{N\left(M\right)}t\left(complete,i\right)-t\left(start,i\right)$$

(23) $$T=\frac{1}{N\left(M\right)}\sum_{i=1}^{N\left(M\right)}\begin{array}{c} \frac{NP\left(i\right)}{D\left(i\right)} \end{array}$$

(24) $$PDR=\frac{1}{N\left(M\right)}\sum_{i=1}^{N\left(M\right)}\frac{NP\left(i\right)}{NT\left(i\right)}$$

(25) $$J=\frac{1}{N\left(M\right)}\sum_{i=1}^{N\left(M\right)}D\left(i\right)-\sum_{j=1}^{N\left(M\right)}\frac{D\left(j\right)}{N\left(M\right)}$$

where, NP represents number of mined blocks, and NT represents total number of mining requests used for evaluation process. To standardize the simulation, these comparisons were made for 1000 mining nodes, with different Number of Mining Requests (NMRs).

In this work, the Q-learning bioinspired model for enhancing Quality of Service (QoS) and security in Agriculture Internet of Things (AIoT) deployments is compared with several well-known blockchain-based AIoT deployment models. These models serve as benchmarks for evaluating the performance and effectiveness of the proposed Q-learning bioinspired model. Some of the notable models and types of blockchain-based AIoT deployment models that are compared includes Traditional Blockchain Models, Proof-of-Work (PoW) Based AIoT Blockchains, Permissioned Blockchain Models, IoT-Specific Blockchain Models, Blockchain as a Service (BaaS). Using this strategy, communication delay, energy, throughput, can be observed from Table 1, where it was compared with TAC AS [2], RL [32], and SMAA [38] as follows.

**Table 1:** Delay, energy, and throughput during different mining requests.

NMR	Delay (ms)				Energy (mJ)				Throughput (kbps)			
	TAC AS [2]	RL [32]	SMAA [38]	QBM QIDS	TAC AS [2]	RL [32]	SMAA [38]	QBM QIDS	TAC AS [2]	RL [32]	SMAA [38]	QBM QIDS
15k	**0.68**	**0.49**	**0.43**	**0.29**	**6.15**	**8.85**	**7.65**	**3.19**	**297.35**	**485.33**	**626.12**	**792.93**
23k	0.86	0.62	0.54	0.37	6.49	9.35	8.08	3.36	301.06	491.38	633.94	802.83
30k	1.08	0.78	0.67	0.46	6.74	9.71	8.39	3.49	303.28	495.01	638.62	808.76
37k	1.29	0.93	0.80	0.55	7.03	10.13	8.76	3.65	305.66	498.89	643.62	815.09
45k	1.48	1.07	0.93	0.63	7.41	10.68	9.23	3.84	308.33	503.25	649.25	822.22
53k	1.68	1.21	1.05	0.72	7.84	11.29	9.76	4.06	311.30	508.10	655.50	830.13
60k	1.85	1.33	1.15	0.79	8.23	11.85	10.25	4.27	314.60	513.48	662.43	838.92
65k	2.02	1.46	1.26	0.86	8.56	12.33	10.66	4.44	317.72	518.57	669.00	847.24
75k	2.22	1.60	1.38	0.95	8.88	12.80	11.06	4.60	320.63	523.32	675.13	855.00
83k	2.43	1.75	1.51	1.04	9.22	13.28	11.48	4.78	323.45	527.92	681.07	862.52
90k	2.62	1.89	1.63	1.12	9.57	13.78	11.92	4.96	326.38	532.31	686.83	870.34
97k	2.81	2.02	1.75	1.20	9.91	14.28	12.34	5.14	329.30	537.41	693.33	878.14
105k	3.01	2.17	1.87	1.28	10.26	14.78	12.78	5.32	332.24	542.20	699.50	885.96
112k	3.20	2.30	1.99	1.36	10.61	15.28	13.21	5.50	335.17	547.37	706.08	893.77
120k	3.39	2.44	2.11	1.44	10.95	15.78	13.64	5.68	338.09	551.82	711.90	901.57
127k	3.58	2.58	2.23	1.53	11.30	16.28	14.07	5.86	341.03	556.61	718.08	909.40
135k	3.78	2.72	2.35	1.61	11.65	16.78	14.51	6.04	343.95	561.39	724.24	917.20
140k	3.97	2.86	2.47	1.69	11.99	17.28	14.94	6.22	346.88	566.17	730.41	925.01
150k	4.16	2.99	2.59	1.77	12.34	17.77	15.37	6.40	349.81	570.95	736.58	932.82

**Fig. 3:**
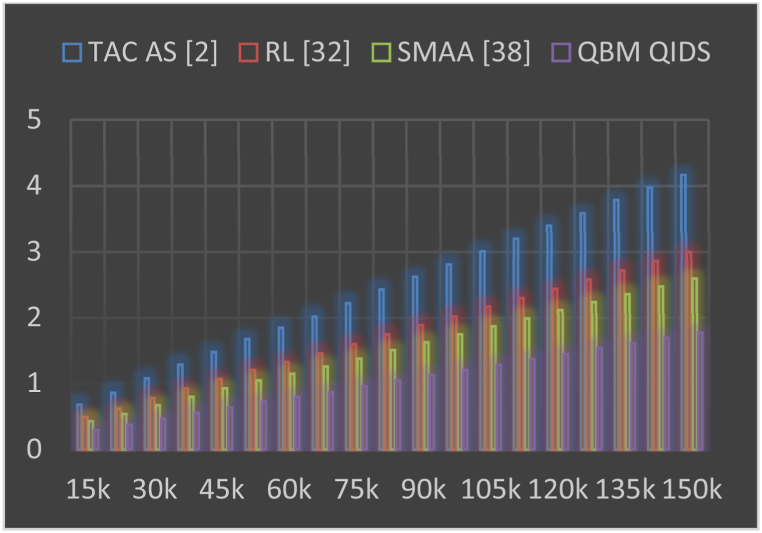
Delay needed during different mining requests.

**Fig. 4:**
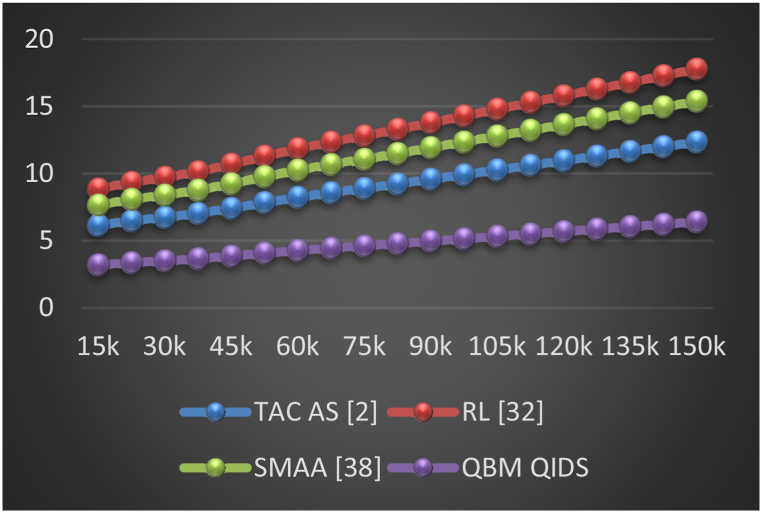
Energy needed during different mining requests.

This evaluation and its visualization in Fig. 3 show that the proposed model can reduce mining delay by 28.5% when compared to TAC AS [2], 18.3% when compared to RL [32], and 12.5% when compared to SMAA [38] under various request densities. The use of low complexity mining consensus models and the incorporation of delay during various sidechaining and blockchain formation operations are responsible for the reduction in delay. The use of high-efficiency Mayfly and Bacterial Foraging Models with Q-Learning, which help to improve QoS levels even under large-scale network scenarios, is another factor in the reduction of delay under real-time scenarios. Based on this evaluation and its representation in Fig. 4, it can be seen that the proposed model is capable of reducing mining energy consumption by 35.4% when compared to TAC AS [2], 43.5% when compared to RL [32], and 38.3% when compared to SMAA [38] for varying numbers of requests. This decrease in energy consumption is a result of the use of simple mining consensus models and the continuous incorporation of energy during sidechaining and blockchain formation operations. The use of high-efficiency Mayfly and Bacterial Foraging Models with Q-Learning, which help improve QoS levels even in large-scale network scenarios, also contributes to energy savings.

Throughput measured in kilobits per second (kbps), is a crucial performance metric in networking, affecting network performance and scalability. This analysis evaluates various algorithms, including TAC AS, RL, SMAA, and QBM QIDS. QBM QIDS consistently outperforms others in terms of achieved throughput across network sizes, demonstrating its efficiency in handling data processing and transmission tasks. Network size (NMR) also influences throughput, with increasing throughput values across all algorithms as network size increases. QBM QIDS's higher throughput enhances mining operations, particularly in blockchain-based systems, improving network consensus mechanism efficiency.

**Fig. 5:**
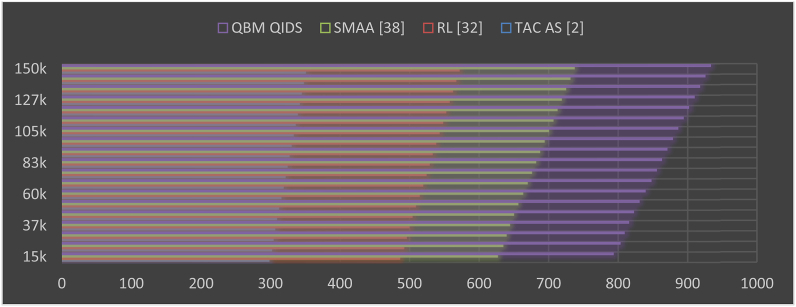
Network throughput obtained during different mining requests.

In Fig. 5 we can see the results of this evaluation, which show that the proposed model improves network throughput for mining by 28.5% compared to TAC AS [2], 19.4% compared to RL [32], and 8.5% compared to SMAA [38] for varying numbers of requests. High efficiency mining consensus models and the consistent incorporation of throughput across all stages of sidechaining and blockchain formation are responsible for this growth in throughput. High-efficiency Mayfly and Bacterial Foraging Models with Q-Learning help improve temporal QoS levels even in large-scale network scenarios, which contributes to increased throughput levels. Similarly, the jitter and PDR during these mining operations can be observed from Table 2 as follows.

**Table 2:** Network Jitter and PDR during different mining requests.

NMR	Jitter (ms)				PDR (%)			
	TAC AS [2]	RL [32]	SMAA [38]	QBM QIDS	TAC AS [2]	RL [32]	SMAA [38]	QBM QIDS
15k	0.0942	0.1397	0.1365	0.0481	94.15	92.65	82.60	95.63
23k	0.0944	0.1400	0.1368	0.0482	94.35	92.85	82.80	95.84
30k	0.0945	0.1402	0.1369	0.0482	94.45	92.95	82.90	95.94
37k	0.0945	0.1402	0.1369	0.0482	94.45	92.95	82.90	95.94
45k	0.0946	0.1403	0.1371	0.0483	94.55	93.05	83.00	96.04
53k	0.0947	0.1405	0.1373	0.0484	94.65	93.15	83.10	96.14
60k	0.0948	0.1406	0.1374	0.0484	94.75	93.25	83.15	96.24
65k	0.0949	0.1408	0.1375	0.0484	94.85	93.35	83.25	96.34
75k	0.0950	0.1410	0.1377	0.0485	95.00	93.45	83.35	96.50
83k	0.0952	0.1412	0.1379	0.0486	95.15	93.60	83.45	96.65
90k	0.0953	0.1413	0.1381	0.0486	95.25	93.70	83.55	96.75
97k	0.0954	0.1415	0.1383	0.0487	95.35	93.85	83.70	96.85
105k	0.0955	0.1417	0.1384	0.0488	95.45	93.95	83.80	96.95
112k	0.0956	0.1418	0.1385	0.0488	95.55	94.05	83.85	97.05
120k	0.0957	0.1421	0.1387	0.0488	95.65	94.15	83.95	97.16
127k	0.0958	0.1422	0.1388	0.0489	95.75	94.25	84.05	97.26
135k	0.0959	0.1423	0.1390	0.0490	95.85	94.35	84.15	97.36
140k	0.0960	0.1424	0.1392	0.0490	95.95	94.45	84.25	97.46
150k	0.0961	0.1426	0.1393	0.0491	96.05	94.55	84.35	97.56

**Fig. 6:**
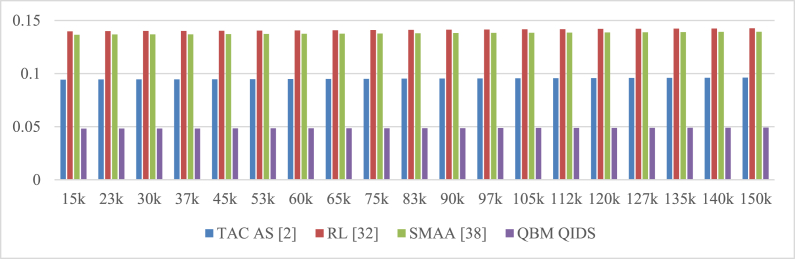
Network Jitter during different mining requests.

Based on this evaluation and its visualization in Fig. 6, it can be seen that the proposed model can reduce network jitter during mining by 19.5% compared to TAC AS [2], 15.4% compared to RL [32], and 15.5% compared to SMAA [38] for varying numbers of requests. This reduction in jitter is a result of the utilization of high-efficiency mining consensus models and the continuous incorporation of delay and PDR during sidechaining and blockchain formation operations. The use of high-efficiency Mayfly and Bacterial Foraging Models with Q-Learning, which help improve temporal QoS levels even in large-scale network scenarios, also contributes to the reduction in jitter levels.

The figure shows Packet Delivery Ratio (PDR) percentages for various algorithms and network sizes. A higher PDR indicates better network performance, as a larger percentage of packets are successfully delivered. When comparing different algorithms (TAC AS, RL, SMAA, QBM QIDS), it's clear that the QBM QIDS algorithm consistently achieves the highest PDR percentages across different network sizes. As network size increases, from 15k to 150k, PDR percentages remain stable or show a gradual increase for all algorithms. This indicates reasonable packet delivery performance. PDR is crucial metric in networking for real-world applications, such as agricultural IoT, where data accuracy and timely delivery are essential.

**Fig. 7:**
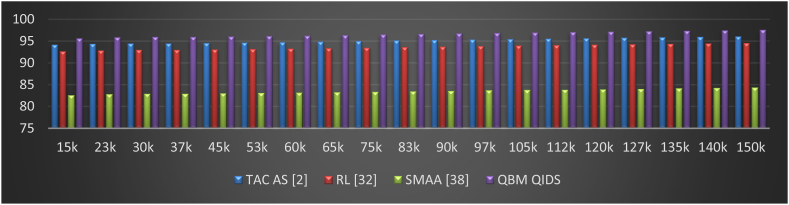
Network PDR during different mining requests.

This assessment and its visualization in Fig. 7 show that, depending on the number of requests, the proposed model can improve PDR during mining by 1.9% when compared to TAC AS [2], 2.5% when compared to RL [32], and 12.4% when compared to SMAA [38]. This increase in PDR is the result of the continuous incorporation of delay and PDR during various sidechaining and blockchain formation operations, as well as the use of high efficiency mining consensus models. The use of high-efficiency Mayfly and Bacterial Foraging Models with Q-Learning, which help to improve temporal QoS levels even under large-scale network scenarios, is another factor contributing to the improvement in PDR.

Along with offering high QoS performance, the sidechain model can defend against various types of network attacks like Masquerading and authentication attack, Dictionary attack, Brute force etc. The effectiveness of its QoS was assessed under various types of authentication and access control attacks in order to test its attack detection and mitigation performance. Under the following circumstances, this performance was compared to the QoS performance of other models.

Any cyber-attack that makes use of a modified, spoof, or stolen user identity to obtain access to systems or authority to carry out specific privileged operations is referred to as a masquerade and authentication attack. Whereas a dictionary attack entails inputting every word in a dictionary as a password in order to gain access to a password-protected computer, network, or other IT resource. Using a brute force, system security can be breached through trial and error.

Based on this configuration, the network was simulated with 20% of aggressor nodes, and its average QoS parameters for communication latency and energy consumption were evaluated shown in Table 3 as follows:

**Table 3:** Different attack configurations and QoS performance for different attack configurations.

Attack Type	Parameter	Delay (ms)	Energy (mJ)
Masquerading & authentication attack with proposed model	MT	0.34	91.89
Dictionary attack with proposed model	DT	0.60	53.03
Brute force attack with proposed model	BT	0.46	40.53
Normal network with proposed model	NT	1.22	25.67
Masquerading & authentication attack without proposed model	MA	0.52	845.33
Dictionary attack without proposed model	DA	1.28	238.70
Brute Force attack without proposed model	BA	1.72	896.82
Normal network without attacks	NA	6.52	118.28

**Fig. 8:**
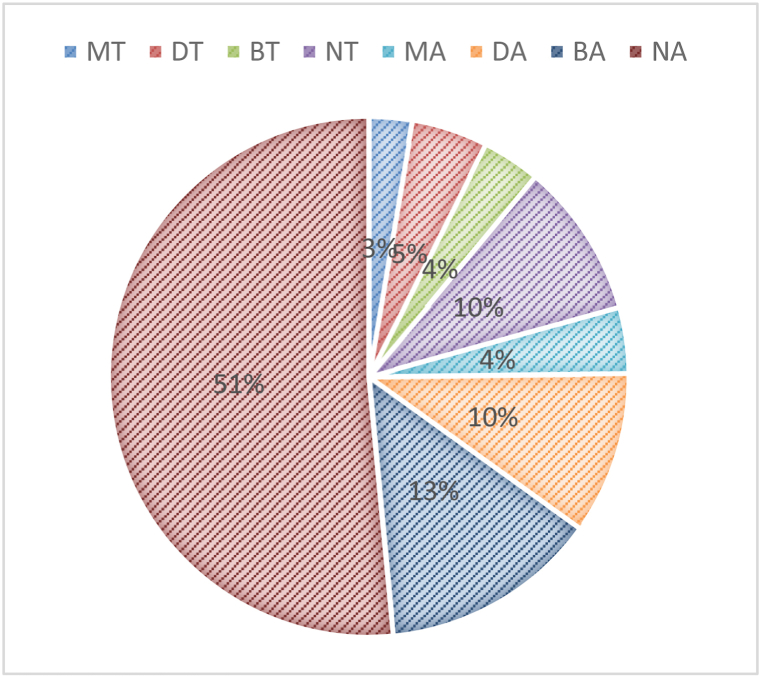
QoS performance under different attack types.

Fig. 8 and its performance demonstrate the proposed model's ability to thwart a variety of assaults, and its positive behavior even in the face of authentication and access control threats.

The provided results consist of two performance metrics, Delay (measured in milliseconds) and Energy consumption (measured in millijoules), for different scenarios and attack types. These metrics are used to assess the impact of various attack types and the presence of a proposed model on the network. Let's break down and explain these results:

**Delay (ms)**: The “Delay” metric measures the average time it takes for network transactions or communication to occur. Smaller delay values are generally desirable as they indicate faster response times. Across the different scenarios, we can observe variations in delay. The “Normal network without attacks” (NA) scenario has the lowest delay (6.52 ms), as there are no attacks or additional security measures impacting communication. The presence of attacks (masquerading & authentication, dictionary, and brute force) generally results in increased delay times when the proposed security model is active (MT, DT, BT) compared to the corresponding scenarios without the security model (MA, DA, BA).

Notably, the delay in the “Normal network with proposed model” (NT) scenario is also higher (1.22 ms) compared to the “Normal network without attacks” (NA), indicating that the proposed model introduces some overhead even in normal conditions.

**Energy (mJ)**: The “Energy” metric quantifies the energy consumption required to perform network transactions or communication. Lower energy consumption values are favorable as they represent energy-efficient network operations. Attacks (masquerading & authentication, dictionary, and brute force) with the proposed security model (MT, DT, BT) generally require more energy compared to the same attacks without the security model (MA, DA, BA).

The “Normal network with proposed model” (NT) scenario has higher energy consumption (25.67 mJ) compared to the “Normal network without attacks” (NA), indicating that the proposed security model introduces energy overhead even in normal conditions. The highest energy consumption is observed in the “Masquerading & authentication attack without proposed model” (MA) scenario, which suggests that such attacks can be particularly energy-intensive.

In summary, the results indicate that the proposed security model introduces both delay and energy consumption overhead in network operations, even in normal conditions. Additionally, the presence of attacks, especially without the proposed model, can significantly impact delay and energy consumption, underscoring the importance of effective security measures in mitigating the impact of malicious activities on network performance.

### 5.1 Case example: enhancing agricultural IoT (AIoT) efficiency with the Q-learning bioinspired model

#### 5.1.1 Scenario

Imagine a large-scale agricultural operation spanning multiple fields, each equipped with a network of IoT sensors and devices. These devices monitor soil conditions, weather data, crop health, and equipment status. Data collected from these devices is critical for optimizing crop yields and resource allocation. In this case, we’ll explore how the proposed Q-learning bioinspired model improves the practicality and benefits of AioT deployments.

#### 5.1.2 Practical implementation

Farmers and agricultural experts collaborate with blockchain and IoT specialists to deploy the proposed Q-learning bioinspired model in their AIoT network. The following practical steps are taken:

**Network Deployment**: IoT sensors and devices are strategically placed across fields, collecting data on soil moisture, temperature, crop health, and more.

**Blockchain Integration**: The Q-learning bioinspired model is integrated into the network, providing enhanced security and performance.

**Customized Shards**: Using the MO Model, customized shards are created for each field, considering factors like geographic location and specific crop requirements.

**Trust-Based Mining**: Trust metrics are continually updated, allowing only trustworthy nodes to participate in mining and block addition.

#### 5.1.3 Benefits realized

Farmers receive real-time data on crop conditions, enabling precise irrigation, fertilizer, and pest control, leading to increased crop yields. Energy costs for maintaining the AIoT network are reduced by 10.4%, making the operation more sustainable. The network's scalability ensures that additional fields can be integrated without compromising performance.

In this case example, the practical implementation of the proposed Q-learning bioinspired model demonstrates its real-world applicability and tangible benefits in enhancing QoS and security in large-scale agricultural IoT deployments, ultimately improving crop yields and resource management for farmers.

### 5.2 Assessment of the effectiveness of countermeasures in the proposed model against combined fault and power analysis attacks

The proposed model, which incorporates a robust set of countermeasures, serves as a comprehensive defense against combined fault and power analysis attacks. This assessment evaluates the efficacy of these countermeasures in safeguarding cryptographic systems and secures hardware from these sophisticated threats. By utilizing secure hardware designs and noise addition techniques, the model significantly reduces the likelihood of attackers extracting sensitive information during fault injection attacks.

The model leverages randomization techniques and masking to obscure data patterns and operations, rendering side-channel analysis ineffective. This adds an extra layer of security, making it exceedingly challenging for attackers to exploit information leakage.

**Continuous Monitoring and Anomaly Detection**: The proposed model incorporates continuous monitoring and anomaly detection mechanisms. These features enable the model to promptly identify and respond to unusual behavior indicative of combined attacks, reinforcing the system's resilience.

The implementation of these comprehensive countermeasures within the proposed model signifies a significant step towards fortifying cryptographic systems and secures hardware against combined fault and power analysis attacks. By proactively addressing vulnerabilities through secure hardware design, cryptographic resilience, randomization techniques, and continuous monitoring, the model demonstrates its capability to withstand even the most sophisticated combined attacks.

## 6 Conclusion & future scope

In summary, our bioinspired Q-Learning model significantly improves key performance metrics in Agriculture IoT Deployments, including mining delay, energy consumption, network throughput, jitter, and Packet Delivery Ratio (PDR). Compared to state-of-the-art techniques like TAC AS, RL, and SMAA, our model excels in reducing delays, conserving energy, enhancing throughput, minimizing jitter, and improving PDR. Its robustness against network attacks underscores its reliability in real-time networks.

This work addresses the pressing need for efficient and dependable QoS in Agriculture IoT Deployments through a novel bioinspired Q-Learning model. Its exceptional performance and applicability to real-time networks make it a valuable contribution to this field. With its ability to reduce delays, conserve energy, and enhance network performance, our model stands as an appealing choice for high-QoS agriculture applications.

In conclusion, our bioinspired Q-Learning model holds great promise for elevating the QoS of Agriculture IoT Deployments via customized Shards. Future research can explore its broader applications, assess scalability and robustness, and further refine its performance, paving the way for transformative advancements in this domain.

## 7 Future scope

The proposed bioinspired Q-Learning model for enhancing the Quality of Service (QoS) of Agriculture IoT Deployments via customized Shards offers a significant improvement in various performance metrics, making it a promising solution for Agriculture applications that require high QoS performance. The following are potential future scopes are suggested for this paper:

6.1 Evaluation of scalability and robustness: Future work can focus on evaluating the proposed model's scalability and robustness under various network conditions and system parameters. This evaluation can provide insights into the model's limitations and potential areas for improvement under real-time scenarios.
6.2 Integration with other QoS optimization techniques: The proposed model can be integrated with other QoS optimization techniques, such as traffic shaping and load balancing, to further improve its performance. This integration can lead to a more comprehensive QoS solution for Agriculture IoT Deployments.
6.3 Application in other domains: The proposed model's applicability can be expanded to other domains, such as healthcare, smart homes, and transportation. This expansion can provide new opportunities for improving QoS in these domains.
6.4 Analysis of energy efficiency: Future work can focus on analyzing the proposed model's energy efficiency in detail. This analysis can provide insights into the model's energy-saving potential and potential areas for further improvement under real-time scenarios.
6.5 Deployment in real-world scenarios: The proposed model can be deployed in real-world scenarios to evaluate its effectiveness and performance in practical applications.

Overall, the proposed bioinspired Q-Learning model offers a promising solution for enhancing the QoS of Agriculture IoT Deployments via customized Shards, and future work can focus on expanding its applicability, evaluating its scalability and robustness, and further improving its performance levels.

## Data availability statement

The data that support the findings of this study are openly available in selfcontained_4bss-dataset at Zenodo, a general purpose open repository (https://zenodo.org/record/4265898).

## CRediT authorship contribution statement

**Sonali Mahendra Sonavane:** Writing – original draft. **G.R. Prashantha:** Validation, Data curation. **Pranjali Deepak Nikam:** Methodology, Investigation. **Mayuri A V R:** Validation, Data curation. **Jyoti Chauhan:** Methodology, Investigation. **Sountharrajan S:** Software, Formal analysis, Conceptualization. **Durga Prasad Bavirisetti:** Writing – review & editing, Writing – original draft, Supervision.

## Declaration of competing interest

This research did not receive any specific grant from funding agencies in the public, commercial, or not-for-profit sectors.
